# Reliability and efficacy of maximum fluorescein tear break-up time in diagnosing dry eye disease

**DOI:** 10.1038/s41598-021-91110-9

**Published:** 2021-06-01

**Authors:** Yujie Mou, Huan Xiang, Lin Lin, Kelan Yuan, Xin Wang, Yaying Wu, Jinjin Min, Xiuming Jin

**Affiliations:** 1 Eye Center, the Second Affiliated Hospital of Zhejiang University, School of Medicine, Hangzhou, China; 2 First People’s Hospital of Wuyi County, Hangzhou, China

**Keywords:** Diseases, Eye diseases, Signs and symptoms, Eye manifestations, Health care, Diagnosis, Quality of life

## Abstract

This study aims to investigate the reliability and efficacy of maximum fluorescein tear break-up time (FTBUTmax) in diagnosing dry eye disease (DED). 147 participants were enrolled in this study. Ocular symptoms were assessed by Ocular Surface Disease Index (OSDI). The fluorescein tear break-up time (FTBUT) examination, corneal fluorescein staining (CFS), and Schirmer I test were performed on both eyes. Each participant underwent 3 consecutive FTBUT tests, and five types of FTBUT values including FTBUTmax, the minimum FTBUT (FTBUTmin), the first FTBUT (FTBUT1), the average of three FTBUTs (FTBUT123) and the average of the first and second FTBUT (FTBUT12) were recorded. FTBUTmax was larger than the other FTBUT values, but no differences were found among the values of FTBUT1, FTBUT123, FTBUT12 and FTBUTmin. In the ROC analysis, FTBUTmax had the largest or the second largest area under the ROC (AUROC) in all three DED diagnostic criteria, while FTBUTmin had the least AUROC of them. ROC efficacy of FTBUTmax was significantly higher than that of FTBUT123, FTBUT12, FTBUT1 and FTBUTmin in the OSDI criteria and higher than that of FTBUT1 and FTBUTmin in *Schirmer I test* and CFS tests. FTBUTmax has a close correlation with OSDI, Schirmer I test and CFS, and is an effective tool for the DED diagnosis.

## Introduction

Dry eye disease (DED) is considered as a complicated ocular disorder, with symptoms affecting 5–30% of the population worldwide^1–3^. The International Dry Eye Workshop (2017) defines DED as “a multifactorial disease of the ocular surface characterized by a loss of homeostasis of the tear film, and accompanied by ocular symptoms, in which tear film instability and hyperosmolarity, ocular surface inflammation and damage, and neurosensory abnormalities play etiological roles”^4^. Several approaches including the staining of cornea and conjunctiva, Shirmer test, tear osmolarity examination and symptomatic questionnaires have been introduced to detect DED^5^, but their reliability and precision in the DED diagnosis is still a challenge. According to previous research, measurement of tear break-up time (TBUT) has been the most widely-adopted dry eye examination because of its convenience and efficacy^6,7^.

TBUT was first introduced by Norn^8^ to assess the stability of tear film. It is traditionally defined as “the interval between the last complete blink and the first appearance of a dry spot or disruption in the tear film”^9^. Over the past decades, fluorescein tear break-up time (FTBUT) has been extensively applied to DED diagnosis by means of a slit-lamp microscope and fluorescence staining. However, its reliability is disputed by different operational and environmental factors, including inter- and intra-observer bias, fluorescein concentration, incomplete blink characteristics, uneven distribution of tear film, air humidity and ambient temperature, etc^10–12^. Still, FTBUT is the mostly welcomed in detecting DED, especially in ophthalmologic-related interdiscipline, such as rheumatic- and gynecologic-related DED, due to the limited use of non-invasive tear break-up time worldwide. Therefore, improvement of old FTBUT methods and exploring new FTBUT statistical approaches are warranted. Instead of using a single reading raised by Norn, Gu^13^ et al. proposed to assess tear film stability and dry eye severity with an average of two or three FTBUT consecutive readings. Interestingly, according to our formal clinical activities, the longest FTBUT (FTBUTmax) among three consecutive measurements appeared to perform well in clinical DED diagnosis and treatment.

To find out which statistical type of FTBUT values is the most applicable to DED diagnosis, five different FTBUT values were raised in our study, namely, the maximum of three consecutive FTBUT values (FTBUTmax), the minimum of three consecutive FTBUT values (FTBUTmin), the first value (FTBUT1), the average of three readings (FTBUT123) and the average of the first and second FTBUT values (FTBUT12). It was found that FTBUTmax could be an effective indicator of DED. Moreover, the efficacy and reliability of FTBUTmax in diagnosing DED was investigated by comparing it with other FTBUT values.

## Materials and methods

### Subjects

This prospective and randomized study was conducted at the outpatient clinics of the Second Affiliated Hospital of Zhejiang University, School of Medicine between October and December 2019, following the tenets of the Declaration of Helsinki. The study was approved by the Ethics Committee of the Second Affiliated Hospital of Zhejiang University School of Medicine. Written informed consent was obtained from each participant after an explanation of the nature and possible consequences of the study.

147 participants from our clinics who were older than 18-year-old voluntarily took part in this study. Patients who had (1) active eye allergies or infections, (2) conjunctival or corneal inflammation, (3) corneal ulcers or scars, (4) eyelid inflammation or movement disorders, (5) palsy, valgus and other ocular lesions, (6) usage of artificial tears within 2 weeks, (7) a history of wearing contact lenses in the preceding 30 days, (8) severe blepharitis, (9) ocular surgeries within 1 year, or (10) severe systemic disease were excluded. For all participants, dry eye examinations and symptomatic analysis were conducted on both right and left eyes.

### Classification and Evaluation of DED

On the occasion that FTBUT values were the study target of our experiment, they could not be used to define and diagnose DED. Therefore, we chose three other parameters for DED detection, i.e., Ocular Surface Disease Index (OSDI), corneal fluorescein staining (CFS) and Shirmer I test. Specifically, an OSDI score > 13 points, a CFS score > 0 point or a Shirmer I test < 10 mm was used as dry eye diagnostic criterion *according to Kim et al*^14^* and Chinese Consensus Guidelines on Dry Eye Disease*^15^*.*

### OSDI

OSDI questionnaires were filled by participants before all dry eye examinations to eliminate the stimulation on corneal sensory estimation. The OSDI is recommended by The International Dry Eye Workshop as a classical and reliable evaluation of DED symptoms. *(Deleted Sentences.)* Patients scoring over 13 would be diagnosed as symptomatic DED^16^.

### FTBUT

FTBUT examination was conducted by an ophthalmologist with more than 20 years of clinical experience. FTBUT values were measured using commercially available sterile fluorescein paper strips (Jinming New Technological Development Co. Ltd., Tianjin, China). Briefly, approximately 5μL (a drop) normal saline was instilled to the trip, which was then shaken to remove extra liquid in order to minimize the volume of fluorescein fluid. Afterwards, the strip was gently touched with the inferior temporal bulbar conjunctiva for 1–2 s. Participants were asked to blink three times naturally to facilitate the uniform distribution of fluorescein on the ocular surface. The time from the last blink of the eye to the first dry spot on the tear film was measured under a cobalt-blue filter. Three consecutive measurements were recorded with a time interval of 30 s. Two eyes were observed separately.

### Corneal fluorescein staining

Corneal fluorescein staining assessment was carried out right after FTBUT testing with the same fluorescein staining strips. Corneal staining was evaluated under a yellow filter using the Oxford scale. Scores over 0 point were regarded as positive.

### Schirmer I test

A sterile 5 mm*30 mm Schirmer strip was gently inserted between the middle and lateral third of each lower lid margin. Participants were then instructed to softly close their eyes. Five minutes later, the length of the wetting strip was recorded in millimeters.

### Statistics

*All statistical analyses were performed with software SPSS for Windows, version 25 (IBM, Chicago, IL, USA) and MedCalc for Windows, version 19 (MedCalc Software, Ostend, Belgium). Figures were created by SPSS and GraphPad Prism, version 8 (San Diego, CA).* Descriptive statistics were summarized as mean ± standard deviation (SD). A Kolmogorov–Smirnov test was used to assess the normality of continuous variables. Raw data of FTBUT undertook logarithmic transformation before statistical analysis. FTBUT values were compared by one-way ANOVA. The receiver operating curve (ROC), area under the ROC (AUROC), cutoff point, sensitivity, specificity and Youden index were calculated. Correlations between FTBUT values and other dry eye parameters were explored using logarithmic Pearson’s correlation coefficient (r). The analysis was double-side and a *p* value < 0.05 was considered statistically significant.

## Results

### Demographics and characteristics

This study included 147 voluntary participants in the outpatient clinics of the Second Affiliated Hospital of Zhejiang University, School of Medicine, including 51 males (34.7%) and 96 females (65.3%) (Table 1). The mean age of all subjects was 37.69 ± 8.73 years. All participants were of Chinese ethnicity. Table 1 shows the basis information of all participants. Results of OSDI, CFS, Schimer I test and five FTBUT statistical types had no significant differences between the right and left eyes. The right eye of each participant was chosen for statistical analysis.

**Table 1:** Demographics of all participants: descriptive statistics.

Parameters	Subjects (n = 147)
Age (year)	37.69 ± 8.73
Gender (male : female)	51: 96
FTBUTmax (second)	6.78 ± 4.48
FTBUTmin (second)	5.73 ± 3.79
FTBUT1 (second)	5.82 ± 4.02
FBUT12 (second)	5.84 ± 4.06
FTBUT123 (second)	5.91 ± 4.33
Corneal fluorescein staining	0.48 ± 0.82
Shirmer I test (mm)	13.26 ± 10.55
OSDI scores	16.84 ± 12.28

Subjects were shown as mean ± standard deviation.

### Comparison of different FTBUT values

**Figure 1:**
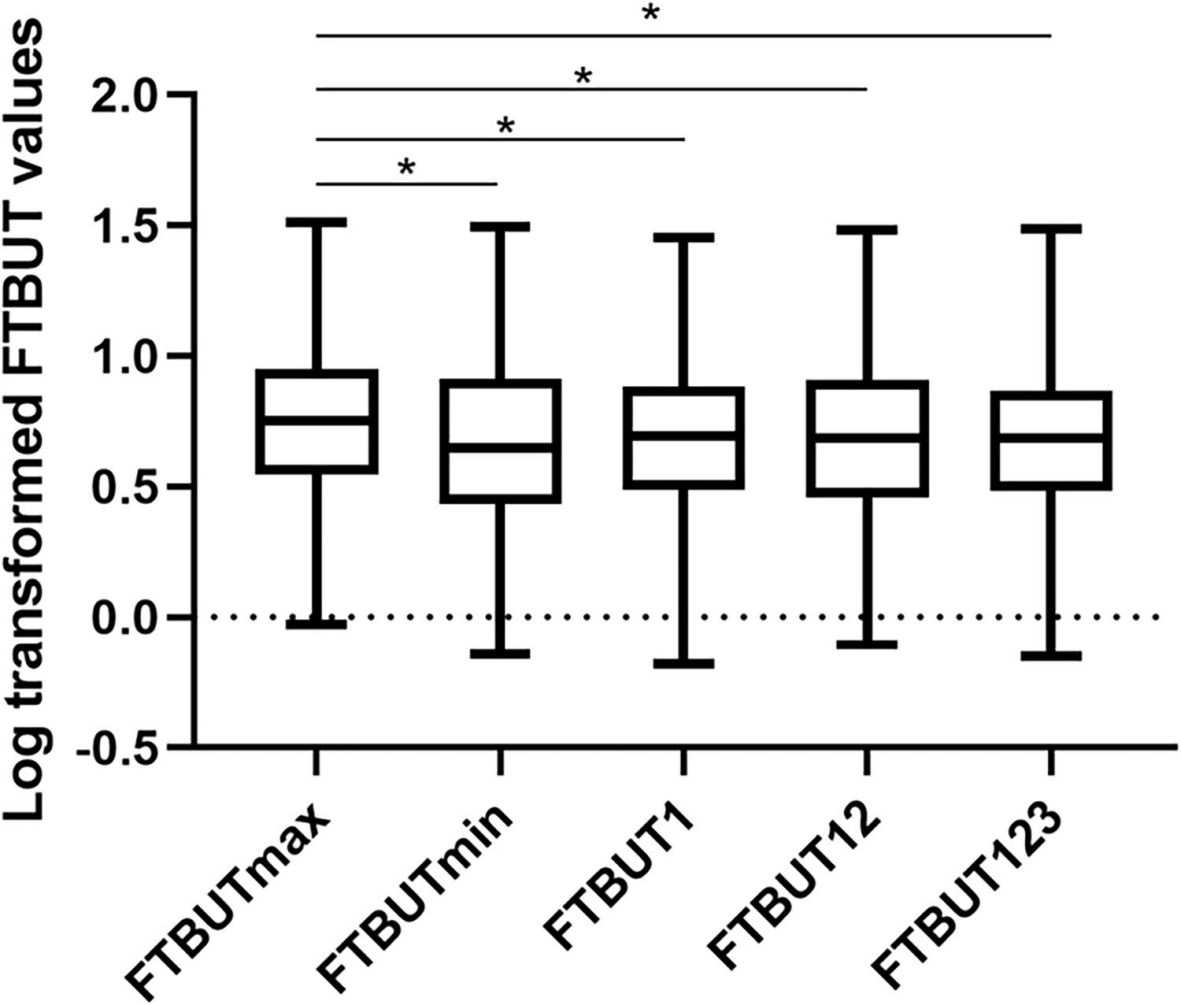
Boxplots of median (95% CI) for the comparison of logarithm transformed FTBUT1, FTBUTmax, FTBUT123, FTBUT12 and FTBUT23 values using one-way ANOVA test. Significant difference was found between FTBUTmax and FTBUT1, FTBUT123, FTBUT12, FTBUT23 respectively (*p* = 0.034, 0.038, 0.040 and 0.025, respectively) while no difference was found among other four FTBUT values. *p < 0.05.

Totally, five FTBUT diagnostic methodologies were analyzed after logarithmic transformation. Statistical differences are demonstrated in Fig. 1. There were statistically significant differences between FTBUTmax and other four FTBUT values, but no significant differences were found among FTBUT1, FTBUT123, FTBUT12 and FTBUTmin.

### ROC curves of five different FTBUT values

**Figure 2:**
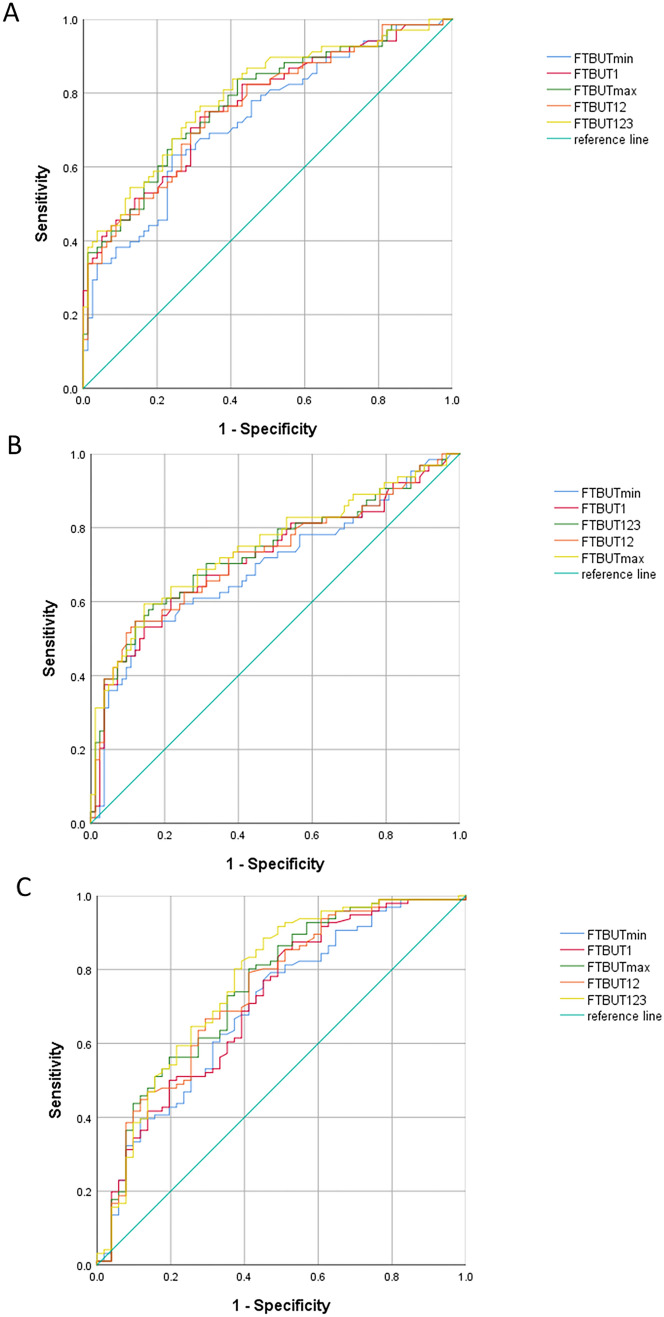
ROC parameters of FTBUTmax, FTBUTmin, FTBUT1, FTBUT12 and FTBUT123 dividing by Schirmer I test (**A**), OSDI scores (B) or CFS (**C**) respectively.

ROC curves and relevant analysis distinctly illustrated the capability and efficacy of each type of FTBUT value in diagnosing DED. The AUROC, cutoff point, sensitivity, specificity and Youden index of FTBUTmax, FTBUTmin, FTBUT1, FTBUT12 and FTBUT123 are displayed in Fig. 2 and Table 2.

**Table 2:** ROC analysis including cutoff point, sensitivity and specificity of different FTBUT values in diagnosing DED.

	FTBUT values	AUROC (95%CI)	Cutoff point(s)	Sensitivity	Specificity	Youden Index
Less than 10 mm in Schirmer I test	FTBUTmax	0.792 (0.717–0.854)	0.6833	0.730	0.726	0.456
	FTBUTmin	0.735 (0.656–0.804)	0.7551	0.608	0.767	0.375
	FTBUT1	0.775 (0.699–0.840)	0.6459	0.743	0.699	0.442
	FTBUT12	0.783 (0.708–0.847)	0.7474	0.743	0.712	0.456
	FTBUT123	0.810 (0.737–0.870)	0.6911	0.716	0.781	0.497
More than 13 points in OSDI score	FTBUTmax	0.747 (0.669–0.815)	0.8404	0.856	0.594	0.449
	FTBUTmin	0.695 (0.614–0.768)	0.8274	0.879	0.531	0.411
	FTBUT1	0.717 (0.637–0.788)	0.8299	0.783	0.609	0.393
	FTBUT12	0.725 (0.645–0.795)	0.8357	0.645	0.795	0.426
	FTBUT123	0.734 (0.626–0.780)	0.8420	0.880	0.550	0.426
More than 0 point in corneal fluorescein staining	FTBUTmax	0.756 (0.673–0.818)	0.5453	0.588	0.802	0.390
	FTBUTmin	0.700 (0.619–0.773)	0.5575	0.529	0.792	0.321
	FTBUT1	0.712 (0.632–0.784)	0.5211	0.471	0.875	0.346
	FTBUT12	0.740 (0.661–0.809)	0.5557	0.588	0.792	0.380
	FTBUT123	0.763 (0.690–0.832)	0.4370	0.549	0.885	0.434

FTBUT values were all undertaken logarithmic transformation.

As indicated in Table 2, in the Shirmer I test, FTBUT123 had the largest AUROC of 0.810 (0.737–0.870), followed by FTBUTmax, whose AUROC was 0.792 (0.717–0.854). FTBUTmin had the smallest AUROC of 0.735 (0.656–0.804) (Fig. 2A). FTBUT123 had a maximal Youden index of 0.497. When adopting the OSDI score as the diagnostic criteria of DED, FTBUTmax had the largest AUROC of 0.747 (0.669–0.815), followed by FTBUT123 [0.734 (0.636–0.780)], and FTBUTmin had the smallest AUROC of 0.695 (0.614–0.768) (Fig. 2B). FTBUTmax had a maximal Youden index of 0.449. In the CFS test, FTBUT123 had the largest AUROC of 0.763 (0.690–0.832), followed by FTBUTmax [0.756 (0.673–0.818)], and FTBUTmin had the smallest AUROC of 0.700 (0.619–0.773) (Fig. 2C). FTBUT123 had a maximal Youden index of 0.434.

### Comparison of ROC curves among five different FTBUT values

The ROC curves of each two types of FTBUT values were compared by Medcalc and their *p* values are shown in Table 3. When dividing by Schirmer I test, ROC efficacy of FTBUTmax was significantly higher than that of FTBUT1 and FTBUTmin, but not different from that of FTBUT123 and FTBUT12. When dividing by OSDI, ROC efficacy of FTBUTmax was significantly higher than that of FTBUT123, FTBUT12, FTBUT1 and FTBUTmin. When dividing by CFS, ROC efficacy of FTBUTmax was significantly higher than that of FTBUT12, FTBUT1 and FTBUTmin, but not different from that of FTBUT123.

**Table 3:** Comparison of *p* values of ROC analysis among FTBUT types.

	Less than 10 mm in Schirmer I test	More than 13 points in OSDI score	More than 1 point in corneal fluorescein staining
FTBUT1-FTBUTmax	0.011*	0.018*	0.004**
FTBUT1-FTBUT123	0.001**	0.125	0.003**
FTBUT1-FTBUTmin	0.002**	0.139	0.350
FTBUT1-FTBUT12	0.337	0.374	< 0.001***
FTBUTmax-FTBUT123	0.006**	0.037*	0.113
FTBUTmax-FTBUTmin	< 0.001***	0.002**	< 0.001***
FTBUTmax-FTBUT12	0.256	0.005**	0.055
FTBUT123-FTBUTmin	< 0.001***	0.006**	< 0.001***
FTBUT123-FTBUT12	0.011*	0.028*	0.049*
FTBUTmin-FTBUT12	0.001**	0.019*	< 0.001***

*p* values were calculated through Medcalc. *p < 0.05, ** *p* < 0.01, ***p < 0.001.

### Correlations between FTBUT values and other dry eye examinations

Pearson’s correlation analysis of logarithmic data and raw data was made. According to Table 4, FTBUTmax, FTBUTmin, FTBUT1, FTBUT12 and FTBUT123 were all closely related to Schirmer I test, the OSDI score and CFS, confirming that FTBUT examinations were reliable.

**Table 4:** Correlations between FTBUT values and other dry eye examinations.

	FTBUT values	R	*p*
Schirmer I test	FTBUTmax-Schirmer I test	− 0.536	< 0.001
	FTBUTmin-Schirmer I test	− 0.463	< 0.001
	FTBUT1-Schirmer I test	− 0.517	< 0.001
	FTBUT12-Schirmer I test	− 0.522	< 0.001
	FTBUT123-Schirmer I test	− 0.556	< 0.001
More than 13 points in OSDI score	FTBUTmax-OSDI	− 0.533	< 0.001
	FTBUTmin-OSDI	− 0.357	< 0.001
	FTBUT1-OSDI	− 0.440	< 0.001
	FTBUT12-OSDI	− 0.450	< 0.001
	FTBUT123-OSDI	− 0.483	< 0.001
More than 0 point in corneal fluorescein staining	FTBUTmax-CFS	− 0.289	< 0.001
	FTBUTmin-CFS	− 0.244	< 0.001
	FTBUT1-CFS	− 0.260	< 0.001
	FTBUT12-CFS	− 0.287	< 0.001
	FTBUT123-CFS	− 0.307	< 0.001

### Distribution of FTBUTmax

The distributions of FTBUTmax among three FTBUT readings are shown in Table 5. The first, second and third FTBUT values were not significantly different (*p* = 0.133). The second FTBUT value turned out to be the largest and the first FTBUT value was the smallest. Intriguingly, among 147 FTBUTmax values, there were 62 s FTBUT values, 54 third FTBUT values and only 31 first FTBUT values.

**Table 5:** Numbers and percentage of each FTBUT reading being the FTBUTmax value.

FTBUT	First FTBUT	Second FTBUT	Third FTBUT
Second(s)	5.73 ± 3.79	5.91 ± 4.43	5.86 ± 4.27
Percentage of being FTBUTmax value (%)	31 (21.1%)	62 (42.2%)	54 (36.7%)

Parameters were presented as mean ± SD.

## Discussion

In this study, the reliability and efficacy of FTBUTmax was investigated by comparing it with other four types of FTBUT values. All five types of FTBUT values were derived from three repeated FTBUT measurements. According to our results, FTBUTmax tended to be a reliable and effective parameter in DED diagnosis on account of its largest or second largest AUROC in three DED diagnostic criteria, including Schirmer I test, the OSDI score and CFS. Besides, the ROC analysis result of FTBUTmax was consistent with the value of Pearson’s correlation coefficient r, which demonstrated its reliability. As for the first FTBUT and minimum FTBUT values, they were biased given the fact that their AUROC values were lower than those of other types of FTBUT values.

Previous studies have doubted about the reliability of FTBUT results since the instillation of fluorescein fluid may reduce the stability of tear film^17,18^. In order to eliminate the negative impact of fluorescein fluid, we shook the wet sodium fluorescein stripe during the test to minimize the volume of fluid instilled into eyes and reduce the irritation of ocular microenvironment^19^. As FTBUT was measured within one or 2 min, the influence of environmental factors could be possibly ignored. Close correlation was found between five FTBUT values (FTBUTmax, FTBUTmin, FTBUT1, FTBUT12 and FTBUT123) and dry eye examinations (Schirmer I test, OSDI and CFS), which verified the reliability of these FTBUT values in measuring the severity and degree of dry eye subjective symptoms.

The patient’s blinking pattern has a great influence on tear film stability as the blinking completion level interferes in the progress of tear film reformation^20^. A complete blink induces the formation of tear film. In this process, the tears from the upper and lower eyelids mix to form an aqueous layer while the Meibomian glands secrete lipids to generate a lipid layer^21^. In incomplete blinking, however, the upper eyelid only partially covers the surface of the eye without making contact with the lower lid, making it impossible for the aqueous layer, lipids, and other secretions to fully expand on the ocular surface. Harrison et al.^22^ found that subjects who often did not complete their blinks experienced a faster rate of tear film break-up and a shorter FTBUT. Our study results implied the first FTBUT value was more likely to be affected by insufficient blinks partly because participants were not familiar with fluorescein examination. It was consistent with the findings of Braun’s study^23^. In addition, the unfamiliarity with FTBUT examination and a lack of compliance may result in unpremeditated movement of participants’ eyeballs and thus less stable FTBUT values. Consequently, ophthalmologists might be deceived by an unstable and biased FTBUT result and provide over-treatment for “patients” who are actually healthy^24^.

Therefore, FTBUTmax, which indicated a relatively full blink as well as a stable ocular environment between blink intervals, proved to be as reliable and effective as FTBUT123 and superior to FTBUTmin, FTBUT1 and FTBUT12 in diagnosing DED. Our study suggests that ophthalmologists announce integrated and precise procedures and matters to patients before FTBUT examination. Several blinks and simulative exercises before the first examination might be useful in reducing the error caused by unfamiliarity.

There are also some limitations in this study. Firstly, a lack of “golden standard” in diagnosing DED made it difficult to identify “real” DED patients. FTBUT values themselves were considered as one of the most vital diagnostic examinations, which rendered their sensitivity and specificity less reliable. Secondly, FTBUT values might be affected by observers and subjects. Camera recording of the FTBUT test and double-blind reading were recommended as they were more precise. However, our study failed to reach the most suitable equipment. To improve the reliability of FTBUT results, we invited a veteran observer with over 20 years of clinical experience. Finally, it was hard to standardize the blink interval of FTBUT. Different lengths of the blink interval and incomplete opening of eyes have complicated impact on the results.

In conclusion, we investigated the performance of a novel FTBUT statistical pattern —— FTBUTmax for tear film assessment in the detection of dry eye. Overall, although the findings of our pilot study require the validation of independent investigations, FTBUTmax seems to be a useful and reliable tool for dry eye diagnosis and the assessment of tear film stability since FTBUT measurement is still widely adopted in clinical practice.
